# Proceedings of the Ohio Dental College Association

**Published:** 1865

**Authors:** J. Taft


### Proceedings of Societies.

### PROCEEDINGS OF THE OHIO DENTAL COLLEGE ASSOCIATION.

The regular annual meeting of the Ohio Dental College Association was held in the lecture room of the college, on Tuesday, February 21st, 1865, at 10 o'clock, A. M.

The President and Vice-Presidents being absent, Dr. Geo. H. Cushing, of Chicago, was chosen chairman, *pro tem.*

The following members were present, viz:

Drs. Geo. H. Cushing, A. Berry, G. W. Keely, G. W. Baxter, S. Wardle, J. Taylor, Geo. E. Jones, J. Taft, W. H. Shadoan, W. P. Horton, J. A. McClelland, J. C. Dean, A. S. Talbert and J Chesebrough.

Minutes of the last meeting were read.

The Treasurer made some remarks in reference to the financial condition of the college, by which it appears that a few shares of stock, are still unsubscribed for, but that no difficulty will be experienced in disposing of these.

The committee appointed at the last meeting to procure new certificates reported that they had attended to the duty assigned them. The new certificates have been obtained and issued to all who have presented their old ones, and to new subscribers, so far as they have paid up their stock.

On motion of Dr. Taylor:

*Resolved,* That Dr. J. A. McClelland of Louisville, Ky., be elected a member of this Association; upon balloting he was unanimously elected.

On motion:

*Resolved,* That a committee of two be appointed to nominate delegates to the American Dental Association, to meet in Chicago, July next.

J. Taft,

J. Chesebrough, } *Committee.*

The following communication was received from the faculty of this Institution:

" *To the Members of the Ohio Dental College Association:*"

We, the undersigned. composing the Faculty of the Ohio College of Dental Surgery hereby respectfully tender to the Trustees of the college, through this Association, our resignations, to take effect at the close of the present session.

H. A. Smith,
Geo. E. Jones,
J. Taft,
J. Taylor,
J. Chesebrough.

On motion:

*Resolved,* That Dr. Taylor be requested to present the resignation of the Faculty to the Board of Trustees, as soon as possible, and request their action upon it at the earliest convenient period.

On motion:

*Resolved,* That a committee of three be appointed, whose duty it shall be to ascertain the best course to be pursued in order to procure a new charter and new organization, with a view of remedying some acknowledged defects, and proceed to the accomplishment of the work, and report to this Association at the earliest moment practicable.

J. TAFT,<br>W. P. HORTON,<br>C. B. CHAPMAN, } *Committee.*

Adjourned till 2 o'clock, P. M.

FIRST DAY.—AFTERNOON SESSION.

The Association met according to adjournment, Dr. Cushing in the chair.

The minutes of the morning session were read and approved.

Dr. Taylor reported that he had notified the President of the Board of Trustees of the resignation of the Faculty, and of the action of this Association, in reference to a new charter and reorganization of the Institution.

REPORT OF THE TREASURER.

The Treasurer would respectfully submit the following report for 1864 to 20th of February, 1865. Cash received from Stockholders as follows:

| | |
|---|---|
| Dr. Wm. Morrison, | $100 00 |
| " S. S. White, | 200 00 |
| " Quinlan, | 100 00 |
| " Honsinger, | 100 00 |
| " Albaugh, | 100 00 |
| " Allport, | 40 00 |
| " Fuller, | 50 00 |
| " A. M. Leslie, | 100 00 |
| " Blake, | 100 00 |
| " Forbes, | 100 00 |
| " Sloan, | 100 00 |
| " Wheeler, | 50 00 |
| " James Leslie, | 100 00 |
| " Geo. H. Cushing, | 100 00 |

| | | |
|---|---|---|
| Dr. | Whitney, | 100 00 |
| " | Hayes, | 100 00 |
| " | Walter, | 50 00 |
| " | Spalding, | 100 00 |
| " | McClelland, | 100 00 |
| " | Horton, | 100 00 |
| " | Jas. Taylor, | 100 00 |
| | | 1,990 00 |

He has paid out the following sums:

| | |
|---|---|
| The balance as per last report, | 35 39 |
| Lifted mortgage, | 1,000 00 |
| Paid express and canceling mortgage, | 4 25 |
| Redeemed college of taxes, | 694 45 |
| Expenses to St. Louis and Chicago, | 50 00 |
| To Cleaveland and Rochester, | 32 00 |
| | 1,816 09 |

On motion:

*Resolved*, That a committee of two be appointed to audit the Treasurer's account:

J. A. McClelland, } *Committee.*

A. S. Talbert. }

The committee appointed in reference to a new charter and reorganization report that they had ascertained that an amendment of the present charter is the most practicable method of proceedure; and under that arrangement, it would be necessary for this Association to nominate nine persons as trustees, under the amended charter. Upon balloting, the following persons were found to be nominated:

Drs. Geo. H. Cushing, Geo. Watt, I. Forbes, James Taylor, A. Berry, H. R. Smith, J. G. Cameron, Geo. W. Keely, and A. S. Talbert.

On motion:

*Resolved*, That the Association now elect officers for the ensuing year.

Upon balloting, the following were found to be elected:

President, J. G. Cameron.

First Vice-President, Geo. H. Cushing.

Second Vice-President, James Taylor.

Secretary, J. Taft.

Treasurer, James Taylor.

On motion:

*Resolved*, That a committee of three be appointed to secure the passage of the amendment of our charter, by the legislature:

J. TAFT,<br>W. P. HORTON,<br>JAMES TAYLOR, } *Committee.*

On motion:

*Resolved*, That the expenses of the committee be paid out of the funds of the Association.

Adjourned till 4 o'clock, P. M., the day following.

SECOND DAY.—WEDNESDAY, 4 O'CLOCK, P. M.

Association met according to adjournment. Minutes of the previous session were read and approved.

Dr. Taylor presented the following report from the Faculty:

REPORT OF THE FACULTY OF THE OHIO COLLEGE OF DENTAL SURGERY.

The Faculty would respectfully report that they have attended to the respective duties assigned them, and that the class for the present session, just expired, was twelve. They are glad to report good attention to studies on the part of the class, and that in mental culture and preparatory education for the profession, the class will compare to advantage with any heretofore in attendance.

The Faculty, adopted, two years since, the cash system, and it has been most strictly enforced this session, and while it has materially lessened the number of the class, yet the effect we consider beneficial on the part of the school.

The Faculty have paid the interest on the $3,000 mortgage, have kept the college insured, have paid the taxes up to June next, and the Institution is out of debt, so far as the Faculty is concerned.

They changed the two front rooms into one, papered and carpeted the same and made such additions to the infirmary as the means placed at their disposal enabled them to do.

JAMES TAYLOR, *Dean.*

WE, the committee appointed by the Ohio Dental College Association to audit the Treasurer's report, have attended to the same, and find it correct. We were also kindly shown the college account, which we find correct.

J. A. MCCLELLAND,<br>A. S. TALBERT, } *Committee.*

The report of the auditing committee was accepted, and the committee discharged.

Dr. Taylor made an explanation in regard to the expenditure of the money subscribed for refitting the infirmary and laboratory, which was quite satisfactory.

On motion of Dr. Leslie:

*Resolved*, That a committee of two, residents of this city, be appointed to procure funds and material for furnishing the laboratory and infirmary with a more perfect outfit, the Faculty to apply the funds.

J. TAFT,<br>A. BERRY, } *Committee,*

Adjourned till to-morrow at 2 o'clock.

THIRD DAY.—AFTERNOON.

The Association met according to adjournment.

Dr. Cushing in the chair. Minutes of the last session were read and approved.

On motion of Dr. Taylor:

*Resolved*, That this Association now proceed to suggest names of persons to fill the chairs in this Institution made vacant by the resignation of the Faculty.

The following were proposed:

Institutes of Dental Science, C. W. SPALDING.

Anatomy and Physiology, C. KEARNS.

Operative Dentistry and Dental Hygeine, J. TAFT.

Chemistry and Therapeutics, GEO. WATT.

Mechanical Dentistry and Metallurgy, H. R. SMITH.

(These nominations to receive the sanction of the Board of Trustees.)

On motion:

*Resolved,* That we recommend two demonstrators in the practical department, one for the infirmary and one for the laboratory, who shall assist and cooperate with the Professors of these respective departments.

On motion:

*Resolved,* That the resolution of the class, just read, be recorded with the minutes of this Association.

It is as follows:

At a meeting of the class held in the lecture room of the college on the 18th inst., the following resolutions were unanimously adopted:

*Resolved,* That the thanks of this class are due and are hereby tendered Dr. William Taft for the kind attention shown us during the absence of Dr. Chesebrough.

*Resolved,* That the thanks of the class are also due Dr. H. R. Smith, for lectures delivered before us.

H. H. Winn,<br>

Frank Slayton,<br>

S. J. Collins, } *Committee.*

On motion of Dr. Berry:

*Resolved,* That Dr. James Taylor be recommended as Emeritus Professor of Institutes of Dental Science.

The committee appointed to select delegates to the American Dental Association reported the following names:

Geo. W. Keely, W. H. Shadoan, Geo. H. Casey, J. G. Cameron, A. Berry, S. Wardle, J. G. Ulrey, J. A. McClelland, J. C. Dean, A. S. Talbert, W. P. Horton, and Geo. Watt.

The minutes were now read and approved.

The Association adjourned to meet on the third Tuesday of February, 1866, at 10 o'clock, A. M.

J. Taft, Secretary.

